# Supplementary material for: The reductive glycine pathway allows autotrophic growth of Desulfovibrio desulfuricans
Source: Nat Commun. 2020 Oct 9;11:5090. doi: 10.1038/s41467-020-18906-7 (PMC7547702; doi:10.1038/s41467-020-18906-7)
Supplement: Supplementary file 1 — Supplementary Information [file 41467_2020_18906_MOESM1_ESM.pdf]

Supplementary Information for: 'The reductive glycine pathway allows autotrophic growth of *Desulfovibrio desulfuricans'* by Sánchez-Andrea *et al.*

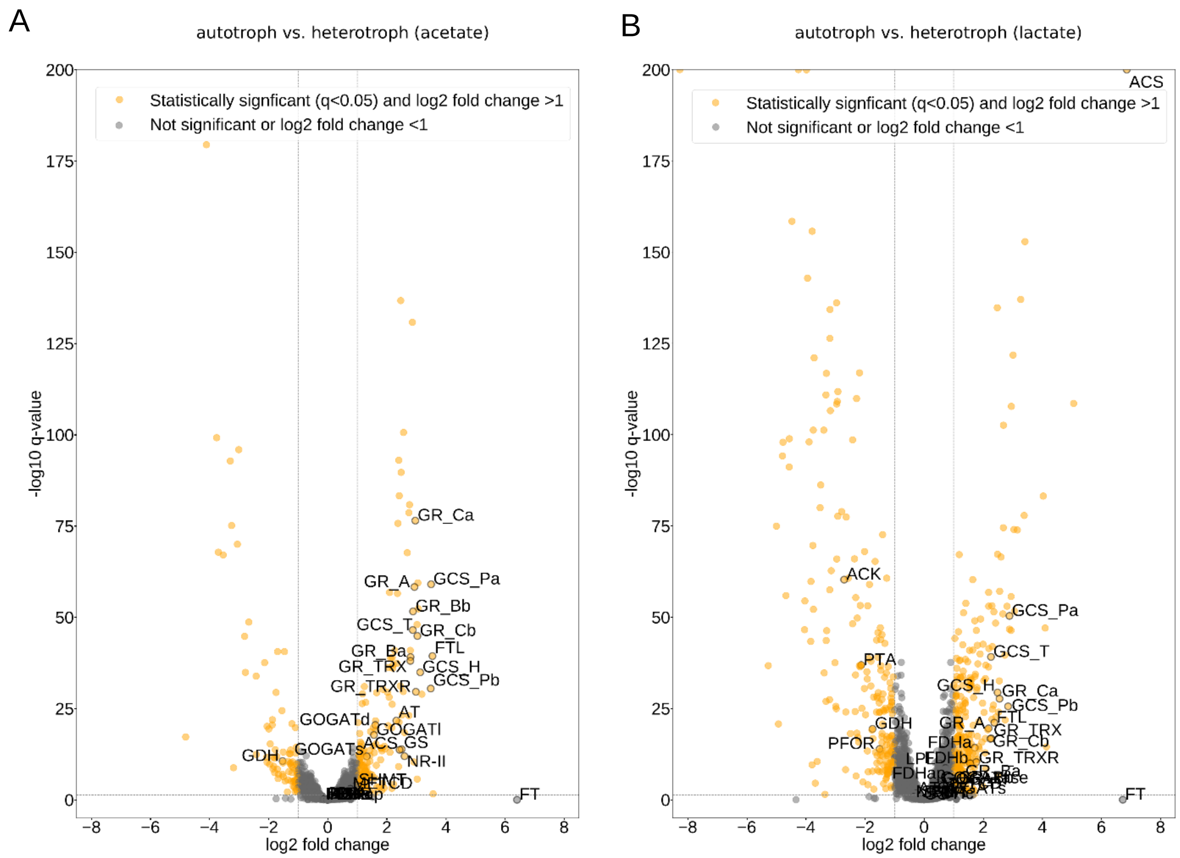

Supplementary Fig. 1. Volcano plots for differential transcriptomics. (A) Transcriptome differences of autotrophic vs. heterotrophic (acetate/H_2/CO_2/sulphate) growth conditions (B) and autotrophic vs. heterotrophic (lactate/CO_2/sulphate) growth conditions. Cultivations were performed in four biological replicates to perform transcriptomic analysis. Growth conditions were 30°C and 175 rpm in 250-ml glass bottles containing 100 ml anoxic minimal medium. FDHa, FDHb, and FDHc (formate dehydrogenase, alpha, beta and gamma subunits); FDHap (formate dehydrogenase, accessory protein); FTL (formate-tetrahydrofolate ligase); METCD (methenyltetrahydrofolate cyclohydrolase); GCS_Pa and GCS_Pb (glycine dehydrogenase/decarboxylating, subunit 1 and 2, glycine cleavage system P-protein); GCS_H (glycine cleavage system H-protein); GCS_T (aminomethyltransferase, glycine cleavage system T-protein); GR_Ba and GR_Bb (glycine reductase complex; component B, subunits beta and alpha); GR_TRXR (thioredoxin-disulfide reductase); GR_Ca and GR_Cb (Glycine reductase complex, component C, subunit alpha and beta); GR_TRX (thioredoxin domain); GR_A (glycine reductase complex, component A); PFOR (pyruvate: ferredoxin oxidoreductase); PTA (phosphate acetyltransferase); ACK (acetate kinase); ACS (acetyl-CoA synthetase); SHMT (serine hydroxymethyltransferase); SDA (serine dehydratase-like); FT (formate transporter); AT (ammonium transporter); NR-II (nitrogen regulatory protein P-II); GS (glutamate-ammonia ligase or glutamine synthetase); GOGATs and GOGATl (glutamate synthase, small and large subunits); GOGATd (glutamine aminotransferase type 2 like domain); GDH (Glutamate dehydrogenase). Source data are provided as a Source Data file, and more extensive transcriptomic data are available in Supplementary Data 4.

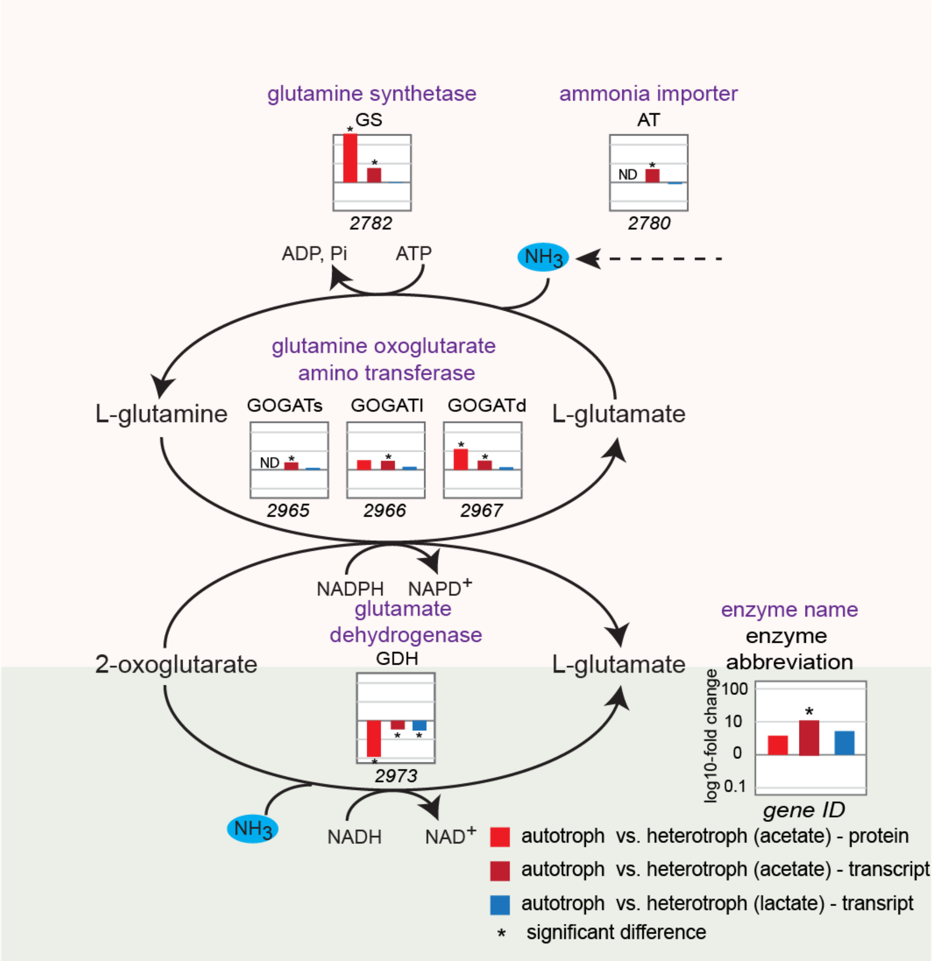

Supplementary Fig. 2. Differential regulation of nitrogen-metabolism genes in *Desulfovibrio desulfuricans* G11. Plots per enzyme represent the log10-fold change in autotrophic condition (H_2/CO_2/sulphate) versus heterotrophic condition (acetate/H_2/CO_2/sulphate) and versus heterotrophic growth on lactate as sole energy source (lactate/CO_2/sulphate), for both proteome and transcriptome analysis. Cultures were performed in four biological replicates to perform proteomic and transcriptomic analysis. Growth conditions were 30°C and 175 rpm in 250-ml glass bottles containing 100 ml anoxic minimal medium. AT (ammonium transporter); NR-II (nitrogen regulatory protein P-II); GS (glutamate-ammonia ligase or glutamine synthetase); GOGATs and GOGATl (glutamate synthase, small and large subunits); GOGATd (glutamine amidotransferase type 2 like); GDH (glutamate dehydrogenase). Source data are provided with this paper, and complete transcriptomics and proteomics data can be found in Supplementary Data 4 and Supplementary Data 5, respectively.

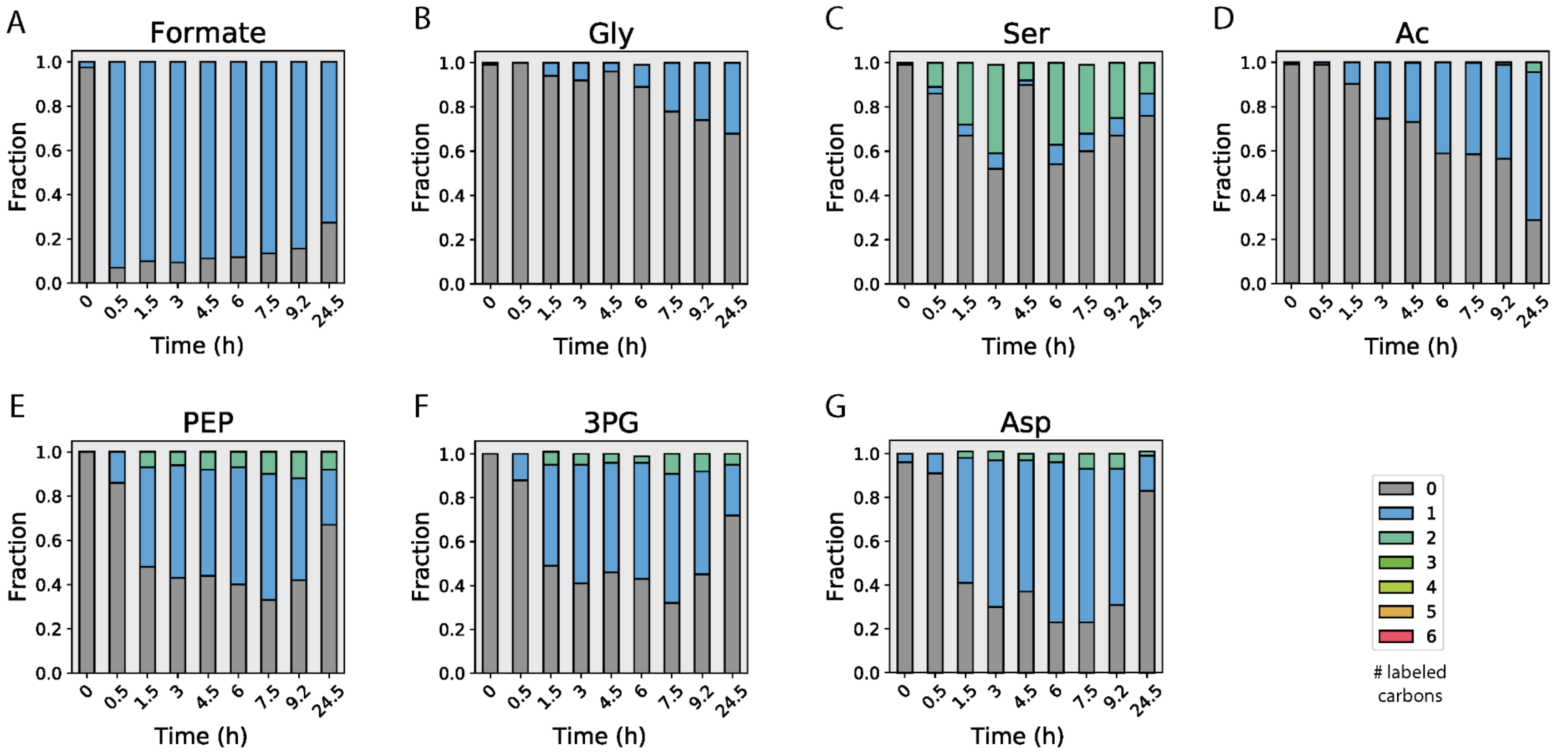

Supplementary Fig. 3. Dynamic labelling experiment of autotrophic growth with ^{13}C-formate. Average mass isotopomer distributions (MID) of selected intracellular metabolites during the 24.5 hour ^{13}C-formate tracing experiment. (A) formate, (B) glycine, (C) serine, (D) acetate, (E) phosphoenolpyruvate, (F) 3-phosphoglycerate, (G) aspartate. Phosphoenolpyruvate and 3-phosphoglycerate are taken as surrogate measurements of pyruvate's MID. Cells were grown in biological triplicates in 1 L glass bottles containing 500 ml anoxic minimal medium at 30C and shaking at 175 rpm. Source data are provided with this paper. A complete list of all measured intracellular and extracellular metabolite MIDs and standard errors can be found in Supplementary Data 6.

Supplementary Table 1: List of genes not identified in the biosynthetic pathways for biomass components in the genome of *Desulfovibrio desulfuricans* G11. Source data are provided with this paper. A complete list of biosynthetic pathways is found in Supplementary Data 2.

Missing gene	EC number	Pathway
Acetylornithine deacetylase	3.5.1.16	Arginine biosynthesis
Phosphoribosyl-ATP diphosphatase	3.6.1.31	Histidine biosynthesis
Homoserine O-acetyltransferase	2.3.1.31	Methionine biosynthesis
Aromatic-amino-acid transaminase	2.6.1.57	Phenylalanine biosynthesis
Tyrosine transaminase	2.6.1.5	Tyrosine biosynthesis
Malate dehydrogenase	1.1.1.37	Reductive TCA cycle
Succinate-CoA ligase (ADP-forming)	6.2.1.5	Reductive TCA cycle
5-amino-6-(5-phospho-D-ribitylamino)uracil phosphatase	3.1.3.104	B2 biosynthesis
2-dehydropantoate 2-reductase	1.1.1.169	B5 biosynthesis
Dihydrofolate reductase	1.5.1.3	B9 biosynthesis
Dihydroneopterin triphosphate diphosphatase	3.6.1.67	B9 biosynthesis
Dihydroneopterin aldolase	4.1.2.25	B9 biosynthesis
5-(carboxyamino)imidazole ribonucleotide synthase	6.3.4.18	IMP biosynthesis
Oleoyl-[acyl-carrier-protein] hydrolase	3.1.2.14	Fatty acid biosynthesis
Glucose-6-phosphate dehydrogenase ($NADP^+$)	1.1.1.49	Pentose Phosphate Pathway
Transaldolase	2.2.1.2	Pentose Phosphate Pathway

Supplementary Table 2: Enzymes, EC numbers and loci tag of all known 6 CO_2 fixation pathways in *D. desulfuricans* G11. The missing genes are highlighted in red. Source data are provided with this paper.

**CO_2 fixation pathways**	**EC number**	**Locus Tag**
	Reductive TCA cycle	
ATP-citrate lyase	EC 2.3.3.8	-
Citrate synthase (for roTCA cycle variant instead of ATP-citrate lyase)	EC 2.3.3.3	DsvG11_1432
Malate dehydrogenase	EC 1.1.1.37	-
Fumarase	EC 4.2.1.2	DsvG11_2369-70
Fumarate reductase	EC 1.3.5.4	DsvG11_2366-68
Succinyl-CoA synthetase	EC 6.2.1.5	-
2-oxoglutarate synthase	EC 1.2.7.3	DsvG11_1472-75
Isocitrate dehydrogenase	EC 1.1.1.42	DsvG11_1431
Aconitase	EC 4.2.1.3.	DsvG11_1429
Pyruvate synthase	EC 1.2.7.1	DSVG11_0940
Pyruvate water dikinase	EC 2.7.9.2	DSVG11_2345
pyruvate carboxylase	EC 6.4.1.1	DsvG11_2344
Malic enzyme	EC 1.1.1.38	DsvG11_2364
	Reductive acetyl-CoA pathway (Wood-Ljungdahl)	
Formate dehydrogenase	EC 1.17.2.3	DsvG11_0566/68/69
Formate tetrahydrofolate ligase	EC 6.3.4.3	DsvG11_3068
Methenyltetrahydrofolate cyclohydrolase	EC 3.5.4.9	DsvG11_1518
Methylenetetrahydrofolate dehydrogenase	EC 1.5.1.15	DsvG11_1518
Methylenetetrahydrofolate reductase	EC 1.5.1.20	DsvG11_1728
Methyltetrahydrofolate:corrinoid methyltransferase	EC 2.1.1.258	-
Carbon monoxide dehydrogenase	EC 1.2.7.4	DsvG11_0759
Carbon monoxide dehydrogenase maturation protein	-	DsvG11_0760

Hydrogenase assembly chaperone	-	DsvG11_0758
Pyruvate:ferredoxin oxidoreductase	EC 1.2.7.1	DsvG11_0940
CODH/ acetyl-CoA synthase	EC 2.3.1.169	-
Archaeal part		
Formylmethanofuran dehydrogenase	EC 1.2.7.12	-
Formylmethanofuran-tetrahydromethanopterin N-formyltransferase	EC 2.3.1.101	-
Methenyltetrahydromethanopterin myclohydrolase	EC 3.5.4.17	-
Methylenetetrahydromethanopterin dehydrogenase	EC 1.5.98.1	-
5,10-methylenetetrahydromethanopterin reductase	EC 1.5.98.2	-
Acetyl-CoA decarbonylase / synthase	EC 2.3.1.-	-
Calvin Cycle		
Ribulose 1,5-biphosphate carboxylase	EC 4.1.1.39	-
Phosphoglycerate kinase	EC 2.7.2.3	DsvG11_0656
Glyceraldehyde-3-phosphate dehydrogenase	EC 1.2.1.12	DsvG11_1466
Triose-phosphate isomerase	EC 5.3.1.1	DsvG11_2943
Fructose-bisphosphate aldolase	EC 4.2.1.13	DsvG11_1465
6-phosphofructokinase/ fructose-bisphosphatase	EC 2.7.1.11/ EC 3.1.3.11	DSVG11_2577/DSVG11_2922
Transketolase	EC 2.2.1.1	DsvG11_0655
Transaldolase	EC 2.2.1.2	-
Fructose-1,6-bisphosphatase class 2/ Sedoheputulose-1,7-bisphosphatase	EC 3.1.3.37	DSVG11_0360
Transketolase	EC 2.2.1.1	DsvG11_0655
Ribulose-phosphate 3-epimerase	EC 5.1.3.1	DsvG11_0654
Ribose 5-phosphate isomerase	EC 5.3.1.6	DsvG11_2548
Phosphoribulokinase	EC 2.7.1.19	-
3-Hydroxypropionate bicycle		

Acetyl-CoA carboxylase biotin carboxyl carrier protein	EC 6.4.1.2	DSVG11_0675-76
Malonyl-CoA reductase	EC 1.2.1.75	-
3-hydroxypropionate dehydrogenase	EC 1.1.1.298	-
Acrylyl-CoA reductase (NADPH) / 3-hydroxypropionyl-CoA dehydratase / 3-hydroxypropionyl-CoA synthetase	EC 6.2.1.36	-
Acrylyl-CoA reductase (NADPH) / 3-hydroxypropionyl-CoA dehydratase / 3-hydroxypropionyl-CoA synthetase	EC 4.2.1.116	-
Acrylyl-CoA reductase (NADPH) / 3-hydroxypropionyl-CoA dehydratase / 3-hydroxypropionyl-CoA synthetase	EC 1.3.1.84	-
(S)-citramalyl-CoA lyase	EC 4.1.3.25	-
3-methylfumaryl-CoA hydratase	EC 4.2.1.153	-
2-methylfumaryl-CoA isomerase	EC 5.4.1.3	-
2-methylfumaryl-CoA hydratase	EC 4.2.1.148	-
Malyl-CoA lyase	EC 4.1.3.24	-
Propionyl-CoA carboxylase	EC 6.4.1.3	-
Methylmalonyl-CoA epimerase	EC 5.1.99.1	-
Methylmalonyl-CoA mutase	EC 5.4.99.2	-
Succinyl-CoA---L-malate CoA-transferase	EC 2.8.3.22	-
Fumarate reductase	EC 1.3.5.4	DsvG11_2366-68
Fumarase	EC 4.2.1.2	DsvG11_2369-70
Malyl-CoA lyase	EC 4.1.3.24	
Dicarboxylate-4-Hydroxybutyrate cycle		
Succinyl-coA reductase	EC 1.2.1.76	-

4-hydroxybutanoate:NADP+ oxidoreductase	EC 1.1.1.-	-
4-hydroxybutyrate---CoA ligase	EC 6.2.1.40	-
4-hydroxybutanoyl-CoA dehydratase	EC 4.1.1.120	-
Enoyl-CoA hydratase	EC 4.2.1.17	-
3-hydroxyacyl-CoA dehydrogenase	EC 1.1.1.35	-
Acetyl-CoA C-acetyltransferase	EC 2.3.1.9	-
Pyruvate synthase	EC 1.2.7.1	DSVG11_0940
Pyruvate water dikinase	EC 2.7.9.2	DSVG11_2345
pyruvate carboxylase	EC 6.4.1.1	DsvG11_2344
Malic enzyme	EC 1.1.1.38	DsvG11_2364
Fumarase	EC 4.2.1.2	DsvG11_2369-70
Fumarate reductase	EC 1.3.5.4	DsvG11_2366-68
Succinyl-CoA synthetase	EC 6.2.1.5	-
3-hydroxypropionate-4-hydroxybutyrate cycle		
Acetyl-CoA carboxylase biotin carboxyl carrier protein	EC 6.4.1.2	DSVG11_0675-76
Malonyl-CoA reductase	EC 1.2.1.75	-
3-hydroxypropionate dehydrogenase	EC 1.1.1.298	-
Acrylyl-CoA reductase (NADPH) / 3-hydroxypropionyl-CoA dehydratase / 3-hydroxypropionyl-CoA synthetase	EC 6.2.1.36	-
Acrylyl-CoA reductase (NADPH) / 3-hydroxypropionyl-CoA dehydratase / 3-hydroxypropionyl-CoA synthetase	EC 4.2.1.116	-
Acrylyl-CoA reductase (NADPH) / 3-hydroxypropionyl-CoA dehydratase / 3-hydroxypropionyl-CoA synthetase	EC 1.3.1.84	-
Propionyl-CoA carboxylase	EC 6.4.1.3	-

Methylmalonyl-CoA epimerase	EC 5.1.99.1	-
Methylmalonyl-CoA mutase	EC 5.4.99.2	-
Succinyl-coA reductase	EC 1.2.1.76	-
4-hydroxybutanoate:NADP+ oxidoreductase	EC 1.1.1.-	-
4-hydroxybutyrate---CoA ligase	EC 6.2.1.40	-
4-hydroxybutanoyl-CoA dehydratase	EC 4.1.1.120	-
Enoyl-CoA hydratase	EC 4.2.1.17	-
3-hydroxyacyl-CoA dehydrogenase	EC 1.1.1.35	-
Acetyl-CoA C-acetyltransferase	EC 2.3.1.9	-

Supplementary Table 3. Comparison of ATP requirements for the biosynthesis of pyruvate for known CO_2 fixation pathways and variants of the reductive glycine pathway. Partly based on [1].

**Pathway**	**Variant**	**ATP equivalents for synthesis of 1 pyruvate**
Reductive acetyl-CoA pathway (Wood-Ljungdahl)		<1[2]
Reductive tricarboxylic acid cycle	Citrate synthase (roTCA)	1
Reductive glycine pathway	Glycine reductase, phosphate acetyl transferase	1
Reductive glycine pathway	Glycine reductase, acetate kinase, acetyl-CoA ligase	2
Reductive glycine pathway	Serine hydroxymethyltransferase, serine deaminase	2
Reductive tricarboxylic acid cycle	ATP-citrate lyase (rTCA)	2
Dicarboxylate-4-hydroxybutyrate cycle		5
3-hydroxypropionate-4-hydroxybutyrate cycle	*Thaumarchaeota*	5
Calvin Cycle		7
3-hydroxypropionate bicycle		7
3-hydroxypropionate-4-hydroxybutyrate cycle	*Crenarchaeota*	9-10[3]

1 Berg, I. A. *et al.* Autotrophic carbon fixation in archaea. *Nature reviews. Microbiology* **8**, 447-460, doi:10.1038/nrmicro2365 (2010).

2 The reductive acetyl-CoA pathway can consume slightly less than one ATP, as some energy is conserved through the generation of transmembrane ion gradients that are coupled to the regeneration of ATP

3 For the 3HP–4HB cycle in the Crenarchaeota phylum, ATP requirements per pyruvate and glycerate-3-phosphate depend on the assimilation pathway used
