# Supplementary material for: The reductive glycine pathway allows autotrophic growth of Desulfovibrio desulfuricans
Source: Nat Commun. 2020 Oct 9;11:5090. doi: 10.1038/s41467-020-18906-7 (PMC7547702; doi:10.1038/s41467-020-18906-7)
Supplement: Supplementary file 3 — Description of Additional Supplementary Files [file 41467_2020_18906_MOESM3_ESM.pdf]

Description of Additional Supplementary Files

File Name: Supplementary Data 1
Description: growth and supernatant analysis of autotrophic, heterotrophic and formatotrophic growth of *D. desulfuricans G11*

File Name: Supplementary Data 2
Description: analysis of annotated metabolic pathways in the genome of *D. desulfuricans G11*

File Name: Supplementary Data 3
Description: analysis genes present and absent for known CO_2 fixation pathways in species related to *D. desulfuricans* G11

File Name: Supplementary Data 4
Description: differential transcriptomics analysis for autotrophic growth versus two modes of heterotrophic growth (acetate/H2/CO2/sulphate and lactate/CO2/sulphate)

File Name: Supplementary Data 5
Description: differential proteomics analysis for autotrophic growth versus heterotrophic growth (acetate/H2/CO2/sulphate)

File Name: Supplementary Data 6
Description: Metabolic analysis data for the 24.5 hour 13C-formate tracing experiment and 13C-formate proteinogenic acid labelling at the end of exponential phase.

File Name: Supplementary Data 7
Description: analysis of the presence of genes encoding the reductive glycine pathway in all available, annotated genomes

File Name: Supplementary Data 8
Description: parameters for genome assembly pipeline
