# Supplementary material for: The reductive glycine pathway allows autotrophic growth of Desulfovibrio desulfuricans
Source: Nat Commun. 2020 Oct 9;11:5090. doi: 10.1038/s41467-020-18906-7 (PMC7547702; doi:10.1038/s41467-020-18906-7)
Supplement: Supplementary file 12 — Reporting Summary [file 41467_2020_18906_MOESM12_ESM.pdf]

nature research

Corresponding author(s): Irene Sanchez-Andrea
Nico Claassens
Alfons Stams

Last updated by author(s): Aug 21, 2020

Reporting Summary

Nature Research wishes to improve the reproducibility of the work that we publish. This form provides structure for consistency and transparency in reporting. For further information on Nature Research policies, see our Editorial Policies and the Editorial Policy Checklist.

Statistics

For all statistical analyses, confirm that the following items are present in the figure legend, table legend, main text, or Methods section.

n/a	Confirmed	
☐	☒	The exact sample size (n) for each experimental group/condition, given as a discrete number and unit of measurement
☐	☒	A statement on whether measurements were taken from distinct samples or whether the same sample was measured repeatedly
☐	☒	The statistical test(s) used AND whether they are one- or two-sided<br>*Only common tests should be described solely by name; describe more complex techniques in the Methods section.*
☒	☐	A description of all covariates tested
☒	☐	A description of any assumptions or corrections, such as tests of normality and adjustment for multiple comparisons
☐	☒	A full description of the statistical parameters including central tendency (e.g. means) or other basic estimates (e.g. regression coefficient) AND variation (e.g. standard deviation) or associated estimates of uncertainty (e.g. confidence intervals)
☐	☒	For null hypothesis testing, the test statistic (e.g. F, t, r) with confidence intervals, effect sizes, degrees of freedom and P value noted<br>*Give P values as exact values whenever suitable.*
☒	☐	For Bayesian analysis, information on the choice of priors and Markov chain Monte Carlo settings
☒	☐	For hierarchical and complex designs, identification of the appropriate level for tests and full reporting of outcomes
☒	☐	Estimates of effect sizes (e.g. Cohen's d, Pearson's r), indicating how they were calculated

Our web collection on statistics for biologists contains articles on many of the points above.

Software and code

Policy information about availability of computer code

Data collection	no special software for data collection was used
Data analysis	NCBI Blast to analyze homology in genes/proteins, genome assembly by smartanalysis pipeline version 2.1.1/SmrtPipe workflow version v1.85.133289; Illumina Trueseq adapters were trimmed from this data with Cutadapt v1.2.1 ; Quality trimming of Illumina sequencing data was performed afterwards with PRINSEQ Lite v0.20.0; The resulting reads were mapped to the PacBio assembly with bowtie2, version 2.2.9; The resulting SAM file was further converted with Samtools, version 1.3.1 ; the assembly was corrected with Pilon version 1.22 ; Non-mapping reads were assembled with IDBA_UD ; Prodigal v2.6.3 was used for prediction of protein coding DNA sequences (CDS) , InterProScan 5.25-64 for protein annotation, Aragorn 1.2.38 for prediction of tRNAs and tmRNA 50 and RNAmmer v1.2 for the prediction of rRNAs 51. CRISPRs were annotated using the CRISPR Recognition Tool v1.2; EC numbers were predicted via PRIAM version March, 2015, and further EC numbers were derived via the GO terms of the InterproScan result. Carbohydrate active enzymes were predicted with dbCAN version 5.0 . The genome was checked with CheckM ; the genome was manually curated and investigated with Pathway Tools; Duplicates were marked with picard tools v.1.124 (http://broadinstitute.github.io/picard/) and SNPs were called with HaploTypeCaller from the GATK package, v4.1.2.0, with --sample-ploidy set to 1. All vcf files were merged with bcftools v1.4 60. ; Identifications of the relevant genes of the reductive glycine pathway in other organisms was performed with tblastx against the NCBI NT database; transcript and read counts were obtained with htseq-count 0.6.1p1; Differential expression analysis was performed with R version 3.5.3 and DESeq2 1.22.2 ; EC numbers of these genes were matched with matplotlib onto the maps of the KEGG database; Filtering and further analysis of the MaxQuant/Andromeda workflow output and the analysis of the abundances of the identified proteins were performed with the Perseus 1.5.5.3 module (available at the MaxQuant suite); LC-MS data for stable isotope labelling were analyzed using Xcalibur (Thermo Scientific, Sunnyvale, CA; dynamic isotope labelling data were analyzed using Maven to obtain extracted ion chromatograms and data was corrected for natural carbon isotope abundances using AccuCor.

For manuscripts utilizing custom algorithms or software that are central to the research but not yet described in published literature, software must be made available to editors and reviewers. We strongly encourage code deposition in a community repository (e.g. GitHub). See the Nature Research guidelines for submitting code & software for further information.

Data

Policy information about availability of data

All manuscripts must include a data availability statement. This statement should provide the following information, where applicable:

- Accession codes, unique identifiers, or web links for publicly available datasets
- A list of figures that have associated raw data
- A description of any restrictions on data availability

All data is available in the main text, supplementary information and data, or public databases. Source data are provided with this paper. The genome sequence of Desulfovibrio desulfuricans G11 and raw genome and transcriptome sequencing data are available are available at ENA under accession number PRJEB22313 [https://www.ebi.ac.uk/ena/browser/view/PRJEB22313]. Proteome data is available in ProteomeXchange under PXD accession number PXD013114 [http://www.ebi.ac.uk/pride/archive/projects/PXD013114].

Field-specific reporting

Please select the one below that is the best fit for your research. If you are not sure, read the appropriate sections before making your selection.

☒ Life sciences ☐ Behavioural & social sciences ☐ Ecological, evolutionary & environmental sciences

For a reference copy of the document with all sections, see nature.com/documents/nr-reporting-summary-flat.pdf

Life sciences study design

All studies must disclose on these points even when the disclosure is negative.

Sample size	Sample sizes for comparative transcriptomics and proteomics were in biological triplicate or quadruplicate (proteomics), which is a generally accepted number of replicates for this type of microbial experiments. Metabolomics were also performed in triplicates
Data exclusions	No data were excluded
Replication	Important experiments (growth experiments, proteomics, transcriptomics, metabolomics) were repeated and well document. No replication issues were reported
Randomization	Randomization was not applied, as bias by the observation order are unlikely in this type of research
Blinding	Blinding was not applied, as by the observer unlikely in this type of research.

Reporting for specific materials, systems and methods

We require information from authors about some types of materials, experimental systems and methods used in many studies. Here, indicate whether each material, system or method listed is relevant to your study. If you are not sure if a list item applies to your research, read the appropriate section before selecting a response.

Materials & experimental systems

n/a	Involved in the study	
☒	☐	Antibodies
☒	☐	Eukaryotic cell lines
☒	☐	Palaeontology and archaeology
☒	☐	Animals and other organisms
☒	☐	Human research participants
☒	☐	Clinical data
☒	☐	Dual use research of concern

Methods

n/a	Involved in the study	
☒	☐	ChIP-seq
☒	☐	Flow cytometry
☒	☐	MRI-based neuroimaging
